# Soluble Triggering Receptor Expressed on Myeloid Cells 1 in lung cancer

**DOI:** 10.1038/s41598-018-28971-0

**Published:** 2018-07-17

**Authors:** Andreas Kuemmel, Astrid Alflen, Lars Henning Schmidt, Martin Sebastian, Rainer Wiewrodt, Arik Bernard Schulze, Roland Buhl, Markus Radsak

**Affiliations:** 1 Department of Hematology, Medical Oncology & Pneumology, University Medical Center Mainz, 55131 Mainz, Germany; 2 Department of Medicine A, Hematology, Oncology and Pulmonary Medicine, University Hospital Medical Center Muenster, 48149 Muenster, Germany; 3 Medical Clinic II, University Hospital Frankfurt, Goethe University Frankfurt, 60590 Frankfurt am Main, Germany

## Abstract

Soluble Triggering Receptor Expressed on Myeloid Cells 1 (sTREM-1) can be found in the sera of patients with infectious, autoimmune and malignant diseases. The primary objective of this study was to investigate the prognostic significance of sTREM-1 in lung cancer patients. We analyzed the sera of 164 patients with lung cancer of all histologies and all stages at the time of diagnosis. We employed an ELISA using the anti-TREM-1 clone 6B1.1G12 mAb and recombinant human TREM-1. Patient data was collected retrospectively by chart review. In ROC-analysis, a sTREM-1 serum level of 163.1 pg/ml showed the highest Youden-Index. At this cut-off value sTREM-1 was a marker of short survival in patients with NSCLC (median survival 8.5 vs. 13.3 months, p = 0.04). A Cox regression model showed stage (p < 0.001) and sTREM-1 (p = 0.011) to indicate short survival. There were no differences in sTREM-1 serum values among patients with or without infection, pleural effusion or COPD. sTREM-1 was not associated with metastasis at the time of diagnosis and was not a predictor of subsequent metastasis. In SCLC patients sTREM-1 levels were lower than in NSCLC patients (p = 0.001) and did not predict survival. sTREM-1 did not correlate with CRP or the number of neutrophils. In non-small cell lung cancer patients, sTREM-1 in serum has prognostic significance.

## Introduction

Triggering Receptor Expressed on Myeloid Cells 1 (TREM-1, CD354) is an innate inflammatory transmembrane receptor first described to be expressed by neutrophils and monocytes^1^. Later, expression has also been reported on various non-immune cells like endothelial cells^2^ and bronchial epithelium^3^. TREM-1 is believed to amplify both infectious and non-infectious inflammation^4^ and to elicit the release of TNF-alpha, IL-8, myeloperoxidase and nitric oxide by innate immune cells^1^. The TREM-1 ligand has not been unequivocally identified so far, but several reports suggest HSP70^5^, HMGB1^6^ and PGLYRP1^7^. On platelets, extracellular actin has recently been suggested as a novel TREM-1 ligand^8^. Moderate expression of TREM-1 during sepsis seems to improve survival in mice, but high expression increases mortality^9^.

During sepsis TREM-1 expression is enhanced and released in a soluble form (sTREM-1). sTREM-1 is a 27 kDa polypeptide consisting of the extracellular domain of TREM-1, that is shed from the cell surface by metalloproteinases (MMP)^10^. In addition, sTREM-1 may be produced as an alternative splicing variant of the TREM-1 mRNA^11^. Initially, sTREM-1 has been proposed to be an accurate marker for infectious diseases such as pneumonia^12^ and sepsis^13^, but later on sTREM-1 in sera has also been found in many non-infectious diseases like COPD^14^, pancreatitis^15^ and inflammatory bowel disease^16^. Hence sTREM-1 can be regarded as a marker for severity of innate inflammation^17,18^. sTREM-1 may act as a kind of decoy receptor for TREM-1 ligands in blood and thus as an anti-inflammatory mediator^19,20^.

In lung cancer, TREM-1 is not expressed by cancer cells, but cancer cells induce the expression of TREM-1 in macrophages^21,22^. These tumor associated macrophages (TAM) may induce a micro-environment promoting tumor growth and nidation of metastatic tumor cells^23,24^. Thus, TREM-1 expression in TAMs is an independent predictor of poor survival in NSCLC^21^.

Corresponding to the supposed anti-inflammatory role of sTREM-1 in inflammation, a study in solid malignancies including lung cancer found sTREM-1 in patients’ sera to be correlated with the absence of metastasis^25^. Contradictory to this, a doctoral thesis found sTREM-1 in sera to be correlated with short survival in lung cancer patients with pleural effusion^26^.

Therefore we conducted the current study to clarify whether sTREM-1 in sera of patients with lung cancer either indicates better survival perhaps by acting anti-inflammatory and preventing metastasis or is an indicator of a fatal inflammatory state leading to shorter survival. Several secondary questions were also addressed: Which cut-off would be used best to discriminate between patients with short and long term survival? Do other diseases known to induce sTREM-1 in sera influence the level of sTREM-1 in lung cancer patients? Does the level of sTREM-1 in sera indicate the presence of pleural effusion or solid metastasis? Can sTREM-1 be used to predict the subsequent occurrence of metastasis in patients treated with curative intention? And finally, is it possible to predict success of anti-cancer therapies by sTREM-1 measurement?

## Results

### sTREM-1 cut-off values

The median level of sTREM-1 in sera was 179.6 pg/ml (minimum 7.7 pg/ml, maximum 1048.3 pg/ml) and the 90^th^ percentile was 361.5 pg/ml. Regarding NSCLC patients only, median level of sTREM-1 was 191.9 pg/ml and 90^th^ percentile was 403.1 pg/ml. In SCLC patients, median level of sTREM-1 was 127.9 pg/ml and 90^th^ percentile was 261.1 pg/ml. None of these cut-off values was predictive for survival in Kaplan-Meier analysis and Cox Regression analysis (data not shown).

**Figure 1:**
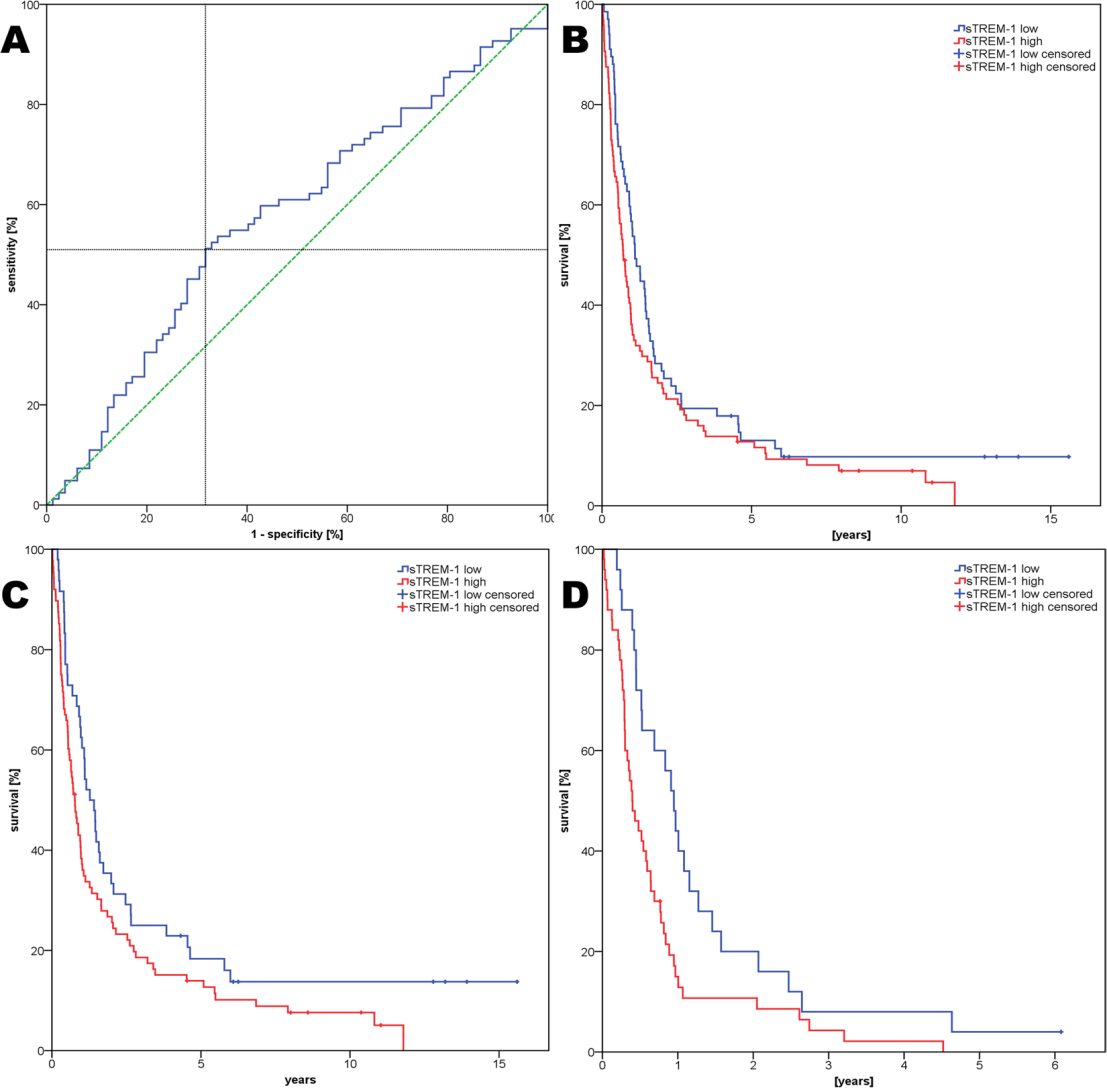
(**A**) ROC-Analysis of sTREM-1 for predicting short survival (the crossing lines mark the highest Youden index at 163.1 pg/ml) (**B**) Kaplan Meier Analysis all patients (n = 164) (**C**) Kaplan Meier Analysis in NSCLC patients (n = 137) (**D**) Kaplan Meier Analysis in stage IV NSCLC patients (n = 75).

We performed a ROC-analysis to determine the level of sTREM-1 predicting survival shorter than the median with the highest Youden’s Index (sensitivity + specificity − 1). Median survival was 10.6 months and only 8.5% of all cases were censored in the study population. The analysis found 3 different levels of sTREM-1 to have an equal Youdens’s Index of 0.195. Of those values, we chose 163.1 pg/ml (42^th^ percentile) as cut-off, because this value displayed the highest specificity and all further survival analysis was done using this cut-off. Sensitivity for detecting a survival shorter than the median was 0.51 and specificity was 0.68 (Fig. 1A).

### Prognostic value in all patients

Patient characteristics of the study population and subpopulations are summarized in Table 1. In the overall study population (n = 164) Kaplan-Meier analysis did not show a prognostic value of sTREM-1 (log rank test p = 0.084, Fig. 1B), but a trend towards shorter survival for patients with sTREM-1 levels higher than 163.1 pg/ml. We performed a multivariate Cox Regression analysis in these patients. The factors age, sex, histology, and sTREM-1 were included in this model. As stage grouping for NSCLC and SCLC were incomparable, we included the presence of metastasis as an additional possible prognostic factor. We found metastasis and sTREM-1 to be of prognostic value (metastasis: p < 0.0001, sTREM-1: p = 0.02) but not sex, age and histology (Table 2).

**Table 1:** Patient characteristics of the study population and subpopulations.

		Study population	Subpopulation				
			A	B	C	D	E
n		164	137	27	62	75	103
Sex (male)		118 (72%)	100 (73%)	18 (67%)	44 (71%)	56 (75%)	76 (74%)
Age (years)		63	63	66	63	62	65
NSCLC		137 (84%)	(100%)	0	(100%)	(100%)	90 (88%)
NSCLC subtype	AC^a^	62 (38%)	62 (45%)		27 (44%)	35 (47%)	45 (44%)
	SCC^b^	56 (34%)	56 (40%)		27 (44%)	29 (39%)	33 (32%)
	AC/SCC^c^	4 (2%)	4 (3%)		4 (7%)	0	2 (2%)
	LCC^d^	4 (2%)	4 (3%)		0	4 (5%)	4 (4%)
	Neuro^e^	2 (1%)	2 (2%)		1 (2%)	1 (1%)	2 (2%)
	Carcinoid	2 (1%–9)	2 (2%)		2 (3%)	0	2 (2%)
	Carc.-sarc.^f^	2 (1%)	2 (2%)		0	2 (3%)	0
	NOS^g^	5 (3%)	6 (4%)		1 (2%)	4 (5%)	2 (2%)
NSCLC stage	I	14 (9%)	14 (10%)		14(23%)		13 (12%)
	II	8 (5%)	8 (6%)		8(13%)		6 (6%)
	III	40(24%)	40(29%)		40(65%)		24 (23%)
	IV	76 (46%)	75 (55%)			(100%)	47 (46%)
SCLC		27 (17%)	0	(100%)	0	0	13 (12%)
SCLC stage	LD	5 (3%)		5 (18%)			3 (3%)
	ED	22 (14%)		22 (82%)			10 (10%)
COPD		n.a.	n.a.	n.a.	n.a.	n.a.	43 (41%)
Pneumonia or sepsis		n.a.	n.a.	n.a.	n.a.	n.a.	13 (13%)

^a^adenocarcinoma, ^b^squamous cell carcinoma, ^c^adenosquamous carcinoma, ^d^large cell carcinoma, ^e^neuroendocrine differentiated carcinoma, ^f^carcinosarcoma, ^g^NSCLC not otherwise specified.

**Table 2:** Cox regression models for overall survival in all patients, in all NSCLC patients and in stage IV NSCLC patients,

study population (n = 164)	HR^b^	FWD^a^ 95% CI^c^	p^d^
Age^e^			0.235
Sex^f^			0.987
Histology^g^			0.099
Metastasis^h^			3.9 × 10^−11^
sTREM-1^i^	1.48	1.06–2.06	0.020
**Subpopulation A (n = 137)**	**HR** ^**b**^	**FWD** ^**a**^ **95% CI** ^**c**^	**p** ^**d**^
Age^e^			0.058
Sex^f^			0.816
sTREM-1^i^	1.6	1.08–2.39	0.011
Stage			4.9 × 10^−11^
1 vs. 2	1.8	0.61–5.17	
2 vs. 3	3.0	1.38–6.64	
3 vs. 4	8.3	3.81–18.01	
Histology			
AC^j^			0.163
SCC^k^			0.388
LCC^l^			0.081
**Subpopulation D (n = 75)**	**HR** ^**b**^	**FWD** ^**a**^ **95% CI** ^**c**^	**p** ^**d**^
Age^e^			0.518
Sex^f^			0.175
sTREM-1^i^	1.94	1.17–3.2	0.008
Histology			
AC^j^			0.193
SCC^k^			0.706
LCC^l^			0.137

^a^Forward likelihood ratio model,

^b^Hazard ratio: HR < 1 suggests improved survival,

^c^Confidence interval,

^d^P-value according to the likelihood ratio test,

^e^Per anno,

^f^Female vs. male,

^g^NSCLC vs. SCLC,

^h^M0 vs M1,

^i^STREM-1 lower vs. higher than 163.1 pg/ml,

^j^O ther NSCLC vs. adenocarcinoma,

^k^Other NSCLC vs. squamous cell carcinoma,

^l^Other NSCLC vs. large cell carcinoma.

### Prognostic value in NSCLC patients

In subpopulation A (all NSCLC patients, n = 137) Kaplan-Meier analysis showed, that patients with a sTREM-1 serum level higher than the cut-off had shorter survival (median survival 8.5 vs. 13.3 months, p = 0.04, Fig. 1C). We performed a multivariate Cox Regression analysis in these patients. The factors age, sex, histology, stage and sTREM-1 were included in this model. We found stage and sTREM-1 to be of prognostic value (stage: p < 0.0001, sTREM-1: p = 0.011) but not sex, age and histology (Table 2).

Regarding stage IV patients (subpopulation D, n = 75), Kaplan-Meier analysis showed, that a high sTREM-1 level was a strong predictor of shorter survival (median survival 4.8 vs. 11.4 months, p = 0.009; Fig. 1D). This was confirmed by a Cox regression model that found a hazard ratio of 1.9 for patients with high sTREM-1level in serum (Table 2).

### Prognostic value in SCLC patients

In subpopulation B (SCLC patients, n = 27), we found a trend towards shorter survival in patients with a sTREM-1 serum level higher than the cut-off (p = 0.07). In the multivariate analysis none of the factors (age, sex, stage and sTREM-1) was accepted.

### Prediction of stage or metastasis in NSCLC patients

**Figure 2:**
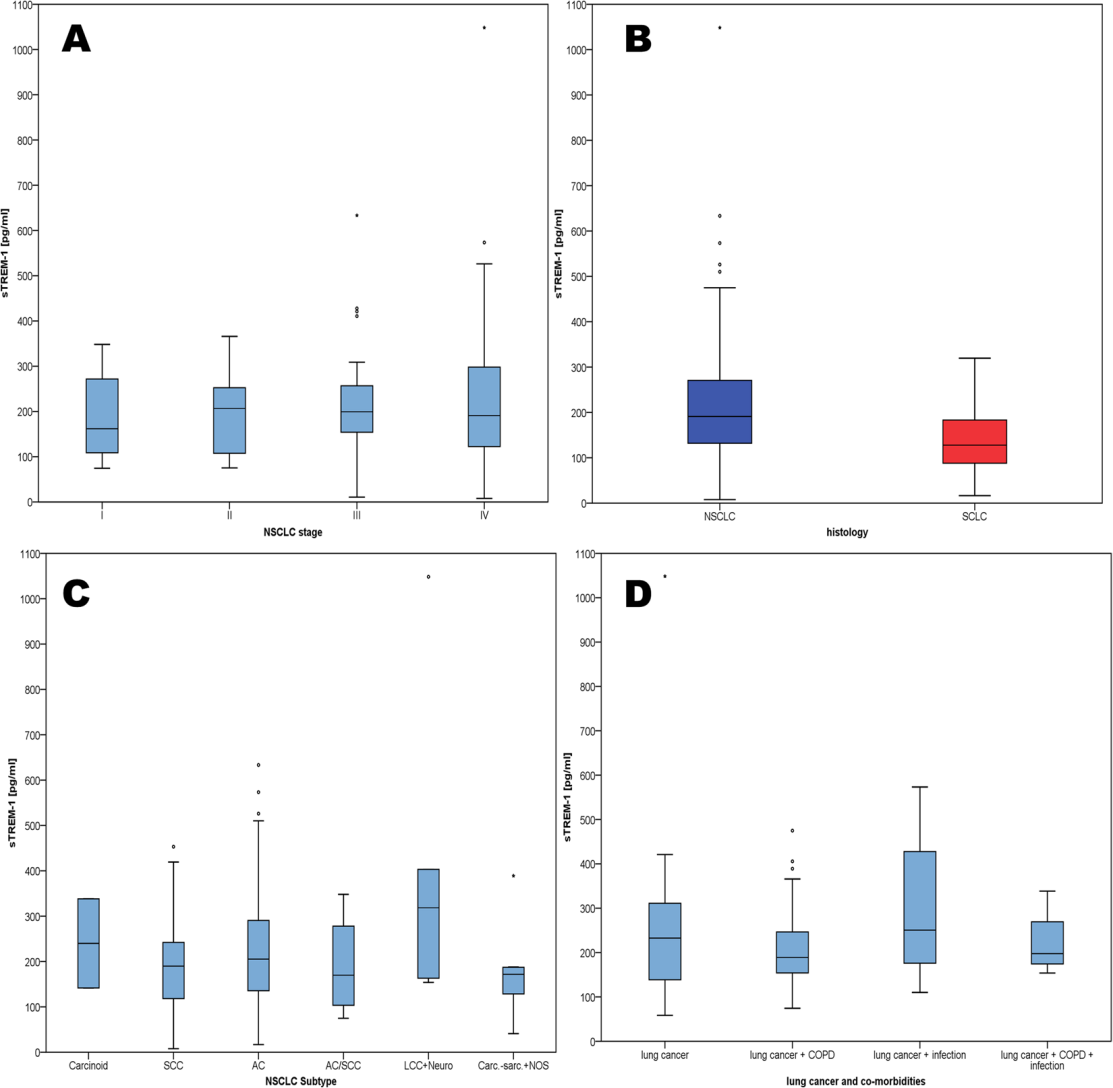
(**A**) Boxplot sTREM-1 in NSCLC stage I vs. II vs. III vs. IV (**B**) Boxplot sTREM-1 in different subtypes of NSCLC (**C**) Boxplot sTREM-1 and Histology (**D)** Boxplot sTREM-1 in lung cancer patients vs. patients with lung cancer and COPD vs. patients with lung cancer and infection vs. patients with lung cancer and infection and COPD).

In a Kruskal-Wallis test, sTREM-1 levels were not different in patients with distinct stages of NSCLC (subpopulation A, p = 0.929; Fig. 2A). There was no association of sTREM-1 with the site of metastasis, especially not with lung metastasis (p = 0.444) at the time of primary diagnosis. In patients with NSCLC stage I-IIIb (subpopulation C), the Kruskal-Wallis test did not show a difference in sTREM-1 values of patients who had a new metastasis after therapy and those who experienced local recurrence during the course of the disease.

### Association of sTREM-1 with histology

There was a significant difference in sTREM-1 between NSCLC and SCLC patients, but not between subtypes of NSCLC (Fig. 2B,C). sTREM-1 serum levels were higher in NSCLC patients (Mann-Whitney U-test p = 0.001). Of the 62 patients with adenocarcinoma, 29 patients had an evaluation of the adenocarcinoma subtype. We found a trend towards higher sTREM-1 levels in patients with acinar adenocarcinoma and bronchioalveolar carcinoma, but the trends did not reach significance in the Mann-Whitney U-test. Of note, when analyzing sTREM-1 values in percentiles, we did not find a patient with SCLC and a sTREM-1 serum level above the 90th percentile (chi square-test p = 0.041), thus very high levels of sTREM-1 could indicate NSCLC.

### Association of sTREM-1 with co-morbidity

In subpopulation E we analyzed, whether there was an association between sTREM-1 values in sera and the presence of co-morbidities like infection (pneumonia or sepsis) or COPD. A Kruskal-Wallis test demonstrated a trend towards higher levels of sTREM-1 in infectious diseases, but the test did not reach significance (p = 0.084; Fig. 2D). In Kaplan-Meier analysis infection was not a predictor of survival (p = 0.520). CRP was reported in 75 patients at the time of blood sampling for sTREM-1. In a linear regression model, sTREM-1 was independent of CRP (R square = 0.088). CRP was associated with infection in Kruskal-Wallis test, but not with survival in a Cox Regression model. There was no correlation of sTREM-1 with LDH (n = 57; R square = 0.05), NSE (n = 108; R square = 0.013), Cyfra (n = 107; R square = 0.015), or CEA (n = 111; R square = 0.005). The total leucocyte count, the percentage of neutrophils and the absolute number of neutrophils (n = 28) did not correlate with sTREM-1 values (R square = 0.05, 0.044 and 0.218).

### Association of sTREM-1 with treatment and response to chemotherapy

Cancer treatment had an impact on sTREM-1 serum levels. 114 Patients (70%) had therapy after sampling for sTREM-1. In 5% of all cases, no treatment data were available. If treatment started prior to the blood sampling, the therapy consisted of chemotherapy in most patients (92%). Regarding NSCLC and SCLC patients, sTREM-1 levels were higher, when the blood sample was taken prior to therapy (Mann-Whitney-U-test p < 0.001), but did not correlate with survival. In the 95 NSCLC patients with a blood test prior to therapy, high sTREM-1 values predicted shorter survival (Kaplan-Meier analysis log rank p = 0.042). We could not confirm these results by multivariate analysis. In NSCLC patients, who had the blood sample after therapy started, we could not observe any difference in survival, but the sample size was low (n = 34). 46 patients had a blood sample before receiving chemotherapy alone for SCLC or NSCLC and had a response evaluation. A Kruskal-Wallis test showed no association of sTREM-1 with the response to chemotherapy (p = 0.724).

## Discussion

The present study demonstrates high sTREM-1 levels in sera as a predictive marker of short survival in NSCLC patients and to be unrelated to the concurrent presence of COPD and infection. NSCLC patients show higher sTREM-1 serum levels than SCLC patients, but we were unable to demonstrate a correlation of sTREM-1 with distinct subtypes of NSCLC, CRP or tumor markers, and there was no association with stage, metastasis or response to chemotherapy.

However, sTREM-1 release might depend on other conditions than infection, non-infectious inflammation or cancer. A recent study^27^ suggests, that the *Trem1* genotype (i.e. the presence of the single nucleotide polymorphism rs2234246) might contribute to higher or lower sTREM1 levels, irrespective of the factors investigated in our study. Moreover, in our patient series, we do not have complete data on the presence of COPD and infection in every patient as the chart review was done retrospectively. Another limitation of our study is the lack of testing for activating EGFR-mutations although it has an important effect on patients’ survival^28^, due to the fact that this was not part of the clinical routine in 2000–2005, when our patients were diagnosed with lung cancer.

The strength of our study is the possibility of studying patients who homogeneously had blood testing for sTREM-1 at the time of first histological diagnosis. Yet, in 25% of our patients therapy was started before sTREM-1 serum testing. These patients had lower levels of sTREM-1 and the impact of sTREM-1 on survival was less evident in this group of patients.

When comparing our results to other studies, it has to be considered, that there are several ELISAs for the detection of sTREM-1 available, which might differ significantly as indicated by the range of cut-off values used in different studies^29^. The ELISA used the study of Hasibeder *et al*.^29^ is most related to our test. We requested data on sTREM-1 levels in healthy volunteers (n = 4) from the authors (personal communication). The median serum level of sTREM-1 in this population was 66.1 pg/ml, which is markedly lower than in our patient population (median level 179.6 pg/ml). Because the numbers were too low for a reliable statistical analysis, we did not perform a Mann-Whitney-U-test or a ROC-analysis.

In a doctoral thesis, Enste analyzed 107 patients with pleural effusion by a comparable ELISA procedure as described in our study^26^. 47 of the patients had lung cancer, 49 had cancer of another primary site and 11 had no malignant disease. Of note, lung cancer patients did not have higher sTREM-1 values in sera than patients without malignancy in that study. The author demonstrated that in the subpopulation of 40 NSCLC patients with pleural effusion (stage III and IV according to UICC 6th TNM Edition) sTREM-1 levels higher than the median of 50 pg/ml correlated with shorter survival. In contrast to our study, the blood sample was taken at an individual time point during that disease, but the results correspond to our finding of sTREM-1 being a negative prognostic marker.

Karapanagiotou *et al*. investigated sTREM-1 in a population of 59 cancer patients including 15 patients with NSCLC and 15 patients with SCLC^25^. In that study, a cut-off value of 15 pg/ml was used. 13% of NSCLC and 33% of SCLC patients exceeded the cut-off which contrasts our finding of higher sTREM-1 serum levels in NSCLC patients.

In the pooled analysis of all cancer patients, Karapanagiotou *et al*. found that high sTREM-1 levels indicated the absence of lung metastasis but not the absence of other metastasis. Obviously, absence of metastasis indicates longer survival in lung cancer. In our study, we could not demonstrate an association with the site of metastasis despite a larger study population. Maybe the effect reported by Karapanagiotou *et al*. is mainly determined by the patients with non-lung cancer entities (breast cancer and colorectal cancer) in their study population. Furthermore the authors use a similar ELISA detection kit as we did in our study, but we lack information if the same capture antibody was used^29^.

In our study, we present the largest analysis of sTREM-1 in sera of lung cancer patients so far. By analyzing the co-morbidities, we can broadly rule out that our main finding of sTREM-1 indicating short survival is confounded by serious infection, a condition that sTREM-1 can also indicate^12^. Our results are in line with the study of Enste^26^ and complement a study of Ho *et al*., who investigated TREM-1 expression in lung cancer tissue by immunohistochemistry^21^.

A possible explanation why patients with high sTREM-1 in serum have a worse prognosis might be that more sTREM-1 is shed from the immune cells surrounding the tumor because of enhanced MMP-activity. MMPs play a role in the degradation of the extracellular matrix during cancer progression and tissue invasion^30^, thus high MMP-activity might indicate a highly aggressive tumor. As we were unable to show an association with tumor stage, this correlation seems insufficient to completely explain the underlying biology. Moreover, we found higher sTREM-1 levels in NSCLC than in SCLC, an entity which is considered to be more aggressive than NSCLC. There are few comparisons of MMPs in SCLC and NSCLC in literature, but MMP-2 seems to be more often expressed on NSCLC cells than on SCLC cells^31,32^. Regarding MMP-9, higher activity in tumor tissues is reported in NSCLC than in SCLC^33^. Potentially, some MMPs might be more relevant for shedding sTREM-1 than others and distinct activity of these MMPs explains our finding of lower sTREM-1 in SCLC. However, this needs to be clarified in future studies.

From the study of Ho *et al*., it is already known that TREM-1 is not expressed on lung cancer cells, but on tumor associated macrophages and that the expression on TAMs could be used to predict short survival in a population of 68 NSCLC patients^21^. Considering that sTREM-1 is the extracellular domain of TREM-1 shed from the cell surface^10^, it would be plausible that high expression of TREM-1 in TAMs lead to high sTREM-1 concentration in serum. In that case, high TREM-1 expression in TAMs and high levels of sTREM-1 in sera could both indicate, that a tumor micro environment has been established that probably promotes tumor growth and therefore signifies an unfavorable course of the disease^24^. In some way contradictory to the study of Ho *et al*.^21^, there is a smaller study which demonstrated less TREM-1 expression in TAMs than in macrophages from normal lung tissue by flow cytometric assays^34^.

Apart from monocytes/macrophages, neutrophils are a major source of sTREM-1. Neutrophils have faster kinetics when shedding sTREM-1 from the cell surface^10^. A study in asthma reported that sTREM-1 levels correlate with neutrophil counts^18^. We did not observe high sTREM-1 levels in patients with high neutrophil numbers in our study.

Recently, checkpoint-inhibitor therapy has fundamentally changed lung cancer treatment. PD-1 is expressed on TAMs^35^ which are believed to play a major role in the interaction of tumor cells with their micro environment, which results in immune evasion^36^. Recent reports suggest that the effect of checkpoint-inhibitor therapy is at least partly mediated by TAMs^37^. In many entities and especially in lung cancer, TAMs seem to polarize to a so called M2 state, which is characterized by CD204 and CD163 expression and promotes tumorigenesis, angiogenesis, remodeling of the extracellular matrix and suppression of immune response^38,39^. In flow cytometric assays, almost all PD-1-positive TAMs express an M2-like surface profile^37^.

In non-malignant disease, hypoxia induced TREM-1-expression on macrophages normally induces a shift from the M2 to the M1 state, which should lead to antimicrobial defense an antitumor resistance^40,41^.

Currently in lung cancer, there are no published studies which investigated how TREM-1 positive TAMs are polarized. A correlation of TREM-1 and the M2 state could explain why the beneficial purpose of TREM-1 in infectious disease gained an opposite role in cancer.

Certainly, our study has the limitation that we are unable to correlate our results with immunohistochemistry using a TREM-1 antibody on the patients‘ tumor specimens as we lack the respective tumor samples. This issue should be addressed in a prospective trial in which blood and tumor samples are available. However, one has to consider that the available sTREM-1 ELISAs differ significantly in precision and accuracy^29^ and need to be improved before thinking of applying sTREM-1 testing in clinical routine.

In conclusion, sTREM-1 may be an interesting surrogate biomarker for TAM activity in NSCLC patients fostering prospective studies investigating whether TREM-1 and PD-1 expression in TAMs both represent the tumor associated M2 state and secondarily whether sTREM-1 can be used as a peripheral blood surrogate marker for this state and potentially as a predictor of checkpoint-inhibitor effectiveness.

## Materials and Methods

We analyzed 164 serum specimens of patients who were referred to our tertiary care center (Pulmonary Division of Gutenberg-University Medical Center, Mainz, Germany) with highly suspected lung cancer or with newly diagnosed lung cancer between 2000 and 2005. Identification of patients, data acquisition and statistical methods were adopted from one of our previous studies in lung cancer^42^. Blood samples were taken within 30 days prior to or after the histopathological diagnosis of lung cancer. The analysis was done in excess material which would otherwise have been disposed after routine diagnostics. The patients gave written consent and the state ethics committee approved this procedure. The samples were immediately centrifuged; sera were aliquoted and frozen at −80 °C. We reviewed the medical records for age, gender, smoking history, co-morbidities, histology, tumor markers (i.e. carcinoembryonic antigen (CEA), cytokeratin fragment 19 (Cyfra), LDH, and neuron-specific enolase (NSE)), clinical staging (according to IUCC/AJCC recommendations including clinical examination, CT scans, bone scan, optional sonography and endoscopy, MRI of the brain if metastasis was suspected or had to be excluded), pathological staging if patients had surgery and therapy data. TNM-staging was based on UICC 6th TNM Edition^43^ and histologic classification was based on the 1999/2004 WHO consensus^44,45^. The study population included patients with non-small cell lung cancer (NSCLC) of stages IA to IV (Classification according to Mountain^46^) and small cell lung cancer (SCLC) patients with limited (LD) or extensive (ED) disease (Veterans’ association^47^). Of note, because patients were staged using UICC 6th TNM Edition, 5 of the 22 patients with pleural effusion but without metastasis were set to have stage III disease. Adenocarcinoma subtypes were only described in 29 of 62 cases based on the WHO classification of tumors published in 1999^45^. Regarding first line therapy, 20 patients (12%) underwent anatomic resection with lymph node dissection. 9 patients (5%) had surgery supplemented by adjuvant or neoadjuvant therapy which involved radiotherapy, chemotherapy or both. In 77 cases (47%) chemotherapy was the only first line therapy. 9 patients (5%) had definitive radiotherapy of the primary tumor and 20 patients (12%) underwent combined radio-chemotherapy. Tyrosine kinase inhibitors (TKIs), monoclonal antibodies or combinations of the former with chemotherapy were administered to 11 patients (7%). Of note, many patients in that group were enrolled in clinical trials and no patient received immune checkpoint-inhibitor therapy. 6 (4%) patients received local therapy of a tumor site other than the primary tumor and moved on to best supportive care. 5 (3%) patients had no therapy other than best supportive care. In 7 cases (5%) data regarding therapy was missing. Medical records were also carefully reviewed for pneumonia or sepsis within 7 day prior or after the serum was analyzed for sTREM-1. If C-reactive protein was measured during this period, the values were also recorded. If a diagnosis of COPD was not known at first admission, pulmonary function test were used to newly diagnose COPD (post-bronchodilatator FEV1/VC < 0.7)^48^. Patient characteristics were summarized in Table 1.

Survival time was calculated from the date of histological or cytological diagnosis to death or last contact with the patient. If the patient was alive on the last contact, the survival time was regarded as censored. A progression of the cancer or recurrent disease assessed by CT scans was observed in 103 patients (63%, deaths not included). 150 patients (92%) died during the follow up period (mean follow-up time: 24 months ± 35 months).

For the detection of soluble TREM-1 (sTREM-1) we used the assay published by Hasibeder *et al*.^29^: 50 μL of anti-TREM-1 (clone 6B1.1G12 mAb) were coated at 10 μg/mL in coating buffer (Na2HPO4 × 2H2O 0.1 M, pH = 9.3) at 4 °C over night and 37 °C for one hour respectively. Then plates were blocked with 200 μL blocking buffer (PBS 1%, BSA 1%) for 1.5 hours at RT. Afterwards the standard (recombinant human TREM-1 in 7.5% BSA-PBS) and the samples were added and the plates were incubated for 1.5 hours at RT. For analysis of sera samples, sera were diluted as indicated prior to addition to the plates (100 μL/well). After incubation for 1.5 hours plates were washed and the biotinylated detection polyclonal Ab anti-TREM-1 (R&D Systems Europe, Abingdon, UK) at 5 μg/mL was added for 1 hour at RT. Plates were then washed and streptavidine-HRP (R&D Systems, Europe, Abingdon, UK) was added for 20 min at RT. Plates were washed again, using the Tetramethylbenzidine Peroxidase Substrate System (KPL, Gaithersburg, Md, USA) and then the reaction was stopped by addition of H2SO4. All dilutions were carried out in blocking buffer. The absorbance was measured at 450 nm.

The results of serologic testing were compared with clinical parameters such as sex, age, CRP, LDH; CEA, Cyfra, NSE, TNM, histology, stage, presence of COPD, sepsis or pneumonia and response to therapy using either Fisher’s exact test or Mann-Whitney U-test. SPSS® 23 software (IBM) was used for the analysis. The median time between diagnosis and the blood sample for sTREM-1 was 6 days. Clinical data was not sufficient to rule out or to rule in sepsis or pneumonia at the time of sTREM-1 testing in 61 cases. CRP was reported in 75 cases. To examine the prognostic value of sTREM-1, we conducted several multivariable Cox proportional hazard regression models using a forward stepwise selection (inclusion criteria: p value of the Score test ≤ 0.05, exclusion criterion: p value of the likelihood ratio test ≥0.1). Model 0 included all patients (n = 164). We chose several subpopulations to answer the studies objectives. Subpopulation A comprised all patients with NSCLC (n = 137) and subpopulation B comprised all SCLC patients (n = 27). The patients with NSCLC stage I-IIIb are summarized in subpopulation C (n = 62). Patients with NSCLC stage IV form subpopulation D (n = 75). Recent data on the presence of sepsis, pneumonia or COPD at the time of diagnosis was available for 103 patients with SCLC and NSCLC. These patients were assigned to subpopulation E.

We considered: stage (1 vs. 2 vs. 3 vs. 4 or limited vs. extensive disease), age (as a continuous variable), sex (male vs. female), NSCLC sub-type (AC vs. other NSCLC, SCC vs. other NSCLC, LCC vs. other NSCLC^44,45^), and sTREM-1 (lower vs. higher than 163.1 pg/ml) as potential prognostic factors (reference category underlined). TNM-Staging^43^ was used in model 0 instead of stage grouping (Mountain^46^ or Veterans’ association^47^) to analyze both SCLC and NSCLC patients (n = 164). For the factors selected by the Cox regression models we compiled univariate Kaplan-Meier charts to visualize the results. We did not determine a global or local level of significance, because we regarded all analyses as explorative. The datasets generated during and/or analyzed during the current study are available from the corresponding author on reasonable request.

## References

[1] Bouchon A, Dietrich J, Colonna M (2000). Cutting edge: inflammatory responses can be triggered by TREM-1, a novel receptor expressed on neutrophils and monocytes. Journal of immunology (Baltimore, Md. : 1950).

[2] Chen LC, Laskin JD, Gordon MK, Laskin DL (2008). Regulation of TREM Expression in Hepatic Macrophages and Endothelial Cells during Acute Endotoxemia. Experimental and molecular pathology.

[3] Rigo I (2012). Induction of triggering receptor expressed on myeloid cells (TREM-1) in airway epithelial cells by 1,25(OH)(2) vitamin D(3). Innate immunity.

[4] Tammaro A (2017). TREM-1 and its potential ligands in non-infectious diseases: from biology to clinical perspectives. Pharmacology & therapeutics.

[5] El Mezayen R (2007). Endogenous signals released from necrotic cells augment inflammatory responses to bacterial endotoxin. Immunology letters.

[6] Wu J (2012). The proinflammatory myeloid cell receptor TREM-1 controls Kupffer cell activation and development of hepatocellular carcinoma. Cancer research.

[7] Read CB (2015). Cutting Edge: identification of neutrophil PGLYRP1 as a ligand for TREM-1. Journal of immunology (Baltimore, Md. : 1950).

[8] Fu L (2017). Identification of Extracellular Actin As a Ligand for Triggering Receptor Expressed on Myeloid Cells-1 Signaling. Frontiers in Immunology.

[9] Gibot S (2007). TREM-1 promotes survival during septic shock in mice. European journal of immunology.

[10] Gomez-Pina V (2007). Metalloproteinases shed TREM-1 ectodomain from lipopolysaccharide-stimulated human monocytes. Journal of immunology (Baltimore, Md. : 1950).

[11] Gingras MC, Lapillonne H, Margolin JF (2002). TREM-1, MDL-1, and DAP12 expression is associated with a mature stage of myeloid development. Molecular immunology.

[12] Gibot S (2004). Soluble triggering receptor expressed on myeloid cells and the diagnosis of pneumonia. The New England journal of medicine.

[13] Gibot S (2005). Surface triggering receptor expressed on myeloid cells 1 expression patterns in septic shock. Intensive care medicine.

[14] Radsak MP (2007). Soluble triggering receptor expressed on myeloid cells 1 is released in patients with stable chronic obstructive pulmonary disease. Clinical & developmental immunology.

[15] Yasuda T (2008). Increased levels of soluble triggering receptor expressed on myeloid cells-1 in patients with acute pancreatitis. Critical care medicine.

[16] Tzivras M (2006). Role of soluble triggering receptor expressed on myeloid cells in inflammatory bowel disease. World journal of gastroenterology.

[17] Tejera A (2007). Prognosis of community acquired pneumonia (CAP): value of triggering receptor expressed on myeloid cells-1 (TREM-1) and other mediators of the inflammatory response. Cytokine.

[18] Bucova M (2012). Inflammatory Marker sTREM-1 Reflects the Clinical Stage and Respiratory Tract Obstruction in Allergic Asthma Bronchiale Patients and Correlates with Number of Neutrophils. Mediators of Inflammation.

[19] Bouchon A, Facchetti F, Weigand MA, Colonna M (2001). TREM-1 amplifies inflammation and is a crucial mediator of septic shock. Nature.

[20] Ford JW, McVicar DW (2009). TREM and TREM-like receptors in inflammation and disease. Current opinion in immunology.

[21] Ho CC (2008). TREM-1 expression in tumor-associated macrophages and clinical outcome in lung cancer. American journal of respiratory and critical care medicine.

[22] Yuan Z (2014). TREM-1 is induced in tumor associated macrophages by cyclo-oxygenase pathway in human non-small cell lung cancer. PloS one.

[23] Takanami I, Takeuchi K, Kodaira S (1999). Tumor-Associated Macrophage Infiltration in Pulmonary Adenocarcinoma: Association with Angiogenesis and Poor Prognosis. Oncology.

[24] Balkwill, F. & Mantovani, A. Inflammation and cancer: back to Virchow? *The Lancet* **357**, 539–545, 10.1016/s0140-6736(00)04046-0. 10.1016/S0140-6736(00)04046-0 11229684

[25] Karapanagiotou EM (2008). Soluble triggering receptor expressed on myeloid cells-1 (sTREM-1) detection in cancer patients: a prognostic marker for lung metastases from solid malignancies. Anticancer research.

[26] Enste, S. R. *sTREM-1 (soluble triggering receptor expressed on myeloid cell-1) im Pleuraerguss*, Johannes-Gutenberg University Mainz (2012).

[27] Aldasoro Arguinano A-A (2017). TREM-1 SNP rs2234246 regulates TREM-1 protein and mRNA levels and is associated with plasma levels of L-selectin. PloS one.

[28] Paez JG (2004). EGFR mutations in lung cancer: correlation with clinical response to gefitinib therapy. Science (New York, N.Y.).

[29] Hasibeder A, Stein P, Brandwijk R, Schild H, Radsak MP (2015). Evaluation and Validation of the Detection of soluble Triggering Receptor Expressed on MyeloidCells 1 by Enzyme-linked immunosorbent Assay. Scientific Reports.

[30] Alaseem, A. *et al*. Matrix Metalloproteinases: A challenging paradigm of cancer management. *Seminars in cancer biology*, 10.1016/j.semcancer.2017.11.008 (2017). 10.1016/j.semcancer.2017.11.008 29155240

[31] Zhang L, Li N, Yan HC, Jiang H, Fang XJ (2017). Expression of Novel CD44st and MMP2 in NSCLC Tissues and Their Clinical Significance. Oncology research and treatment.

[32] Michael M (1999). Expression and Prognostic Significance of Metalloproteinases and Their Tissue Inhibitors in Patients With Small-Cell Lung Cancer. Journal of Clinical Oncology.

[33] El-Badrawy MK, Yousef AM, Shaalan D, Elsamanoudy AZ (2014). Matrix metalloproteinase-9 expression in lung cancer patients and its relation to serum mmp-9 activity, pathologic type, and prognosis. Journal of bronchology & interventional pulmonology.

[34] Zhang G (2016). TREM-1low is a novel characteristic for tumor-associated macrophages in lung cancer. Oncotarget.

[35] Zhang Y, Du W, Chen Z, Xiang C (2017). Upregulation of PD-L1 by SPP1 mediates macrophage polarization and facilitates immune escape in lung adenocarcinoma. Experimental cell research.

[36] Quatromoni JG, Eruslanov E (2012). Tumor-associated macrophages: function, phenotype, and link to prognosis in human lung cancer. American journal of translational research.

[37] Gordon SR (2017). PD-1 expression by tumour-associated macrophages inhibits phagocytosis and tumour immunity. Nature.

[38] Komohara Y, Jinushi M, Takeya M (2014). Clinical significance of macrophage heterogeneity in human malignant tumors. Cancer science.

[39] Hirayama S (2012). Prognostic impact of CD204-positive macrophages in lung squamous cell carcinoma: possible contribution of Cd204-positive macrophages to the tumor-promoting microenvironment. Journal of thoracic oncology: official publication of the International Association for the Study of Lung Cancer.

[40] Raggi F (2017). Regulation of Human Macrophage M1-M2 Polarization Balance by Hypoxia and the Triggering Receptor Expressed on Myeloid Cells-1. Front Immunol.

[41] Subramanian S, Pallati PK, Sharma P, Agrawal DK, Nandipati KC (2017). TREM-1 associated macrophage polarization plays a significant role in inducing insulin resistance in obese population. Journal of translational medicine.

[42] Kuemmel A (2015). Humoral immune responses of lung cancer patients against the Transmembrane Phosphatase with TEnsin homology (TPTE). Lung cancer (Amsterdam, Netherlands).

[43] Greene, F. *et al*. AJCC Cancer Staging Manual, 6th edn Springer: New York. Ref Type: Report (2002).

[44] Travis, W. D., Brambilla, E., Mller-Hermelink, H. K. & Harris, C. C. In World Health Organization Classification of Tumours. Pathology and Genetics. Tumours of the Lung, Pleura, Thymus Vol. 1 9–122 (IARC Press: Lyon 2004, 2004).

[45] Travis, W. D. *et al*. *Histological Typing of Lung and Pleural Tumours*. (Springer Verlag Berlin Heidelberg, 1999).

[46] Mountain CF (1997). Revisions in the International System for Staging Lung Cancer. Chest.

[47] Green RA, Humphrey E, Close H, Patno ME (1969). Alkylating agents in bronchogenic carcinoma. The American journal of medicine.

[48] Vogelmeier CF (2017). Global Strategy for the Diagnosis, Management, and Prevention of Chronic Obstructive Lung Disease 2017 Report. GOLD Executive Summary. American journal of respiratory and critical care medicine.

